# Refractory Ascites in Patients With Cirrhosis

**DOI:** 10.1002/jgh3.70245

**Published:** 2025-07-31

**Authors:** Madhumita Premkumar

**Affiliations:** ^1^ Department of Hepatology Post Graduate Institute of Medical Education and Research Chandigarh India

**Keywords:** albumin, liver transplantation, paracentesis, refractory ascites, TIPS, transjugular intrahepatic portosystemic shunt

Recurrent ascites refers to fluid accumulation in the abdomen that returns at least three times per year, despite dietary sodium restriction and diuretic treatment. It may precede the development of refractory ascites (RA). RA is characterized by ascites that cannot be resolved or whose early recurrence (after large volume paracentesis [LVP]) is not prevented by medical therapy [1]. RA is typically classified as either diuretic resistant or diuretic intolerant.

Management of RA includes ongoing sodium restriction with regular monitoring, frequent LVP, ≥ 5 L combined with infusion to prevent paracentesis‐induced circulatory dysfunction, and possibly albumin infusions outside of paracentesis. For RA patients who do not respond to diuretics or experience significant side effects at maximum doses, alternative treatments should be considered, as further use increases complications without benefit. In the ANSWER study, Caraceni et al. [2] reported that long‐term human albumin therapy improved overall survival in cirrhosis patients with uncomplicated ascites compared to standard treatment. Tolvaptan has been used as adjunctive therapy in patients with hyponatremia and RA [3, 4]. In suitable patients with preserved liver function, a covered, smaller‐diameter transjugular‐intrahepatic portosystemic shunt (TIPS) can improve quality of life and survival post‐ascites clearance. Patients with RA are also likely to have recurrent episodes of hepatorenal syndrome‐acute kidney injury (HRS‐AKI) [5] and also cirrhotic cardiomyopathy (CCM) [6], which in turn impairs the health‐related quality of life (HRQoL). For patients with RA who cannot undergo liver transplantation (LT) or TIPS, LVP and albumin infusion are the only treatments [7]. Future options may include automated low‐flow ascites pumps [8].

LVPs are often needed weekly or fortnightly, straining hospital resources and causing unplanned admissions that reduce quality of life (QoL) and increase costs. Also, LVPs require point‐of‐care ultrasound guidance [9] to minimize the risks of bleeding [10] and infection.

Regular home drainage could prevent these frequent hospitalizations. As ascites drainage is palliative in patients unsuitable for LT, it should follow palliative care principles [11]. Indwelling catheters, commonly used in malignant ascites and hydrothorax, offer a viable care model.

In this issue of JGHOpen, Ramachandran et al. [12] describe the palliative impact of long‐term abdominal drain (LTAD) insertion in patients with RA. Fifty‐one cirrhosis patients with RA were screened; 7 underwent LT, 6 chose TIPS, and 12 had drains inserted for analysis. Six deaths occurred; none related to LTAD. All participants preferred LTAD over LVPs and were highly satisfied. Of the four who completed 6 months, three continued LTAD; the fourth had improved liver function after abstaining from alcohol. Drains carry a significant risk of complications—two cases of spontaneous bacterial peritonitis occurred: one due to missed antibiotics, the other from repeated drain adjustments. Both infections resolved with IV antibiotics without removing the LTAD. Local complications, including cellulitis and leakage, were mild and non‐serious.

Patients with RA should be evaluated for liver transplantation (LT) given the associated poor prognosis. In a randomized controlled trial conducted by Macken et al. [13], 36 patients were assigned to either long‐term abdominal drain (*n* = 17) or large‐volume paracentesis (LVPs, *n* = 19). The incidence of self‐limiting cellulitis or leakage was 41% (7/17) in the long‐term abdominal drain group compared to 11% (2/19) in the LVP group. Peritonitis occurred in 6% (1/17) of patients in the long‐term drain group versus 11% (2/19) in the LVP group. Again, it is unclear if long‐term albumin therapy should be used in patients with LTAD [14].

Key unresolved issues include optimal long‐term albumin use, beta‐blocker application, ideal TIPS timing, and best stent diameter for minimizing shunt‐related side effects in ascites management [15]. Lastly, patients often receive SBP prophylaxis with mixed results. In the current study, prescribing antibiotics as primary prophylaxis to all trial participants to prevent SBP is also not supported based on updated evidence [16]. Long‐term use of antibiotics presents risks to individuals and the wider population, increasing antimicrobial resistance, so future trials should thoughtfully consider this strategy [17]. The management of ascites should therefore be personalized to suit the goals of care for a patient [18]. Other medications that have been used in small studies include the use of midodrine for elevating the mean arterial pressure and improving diuretic response [19] and the use of Sodium glucose cotransporters to improve diuresis by augmenting glycosuria. Patients with CCM and diastolic heart failure may benefit from the addition of drugs like empagliflozin, which has been shown to improve control of ascites in small studies and has ameliorated cardiac dysfunction through pleiotropic effects [20]. Figure 1 shows the current need for personalized management of patients with RA. The best result will be to perform a liver transplantation, but it may not be feasible in resource‐constrained settings.

**FIGURE 1:**
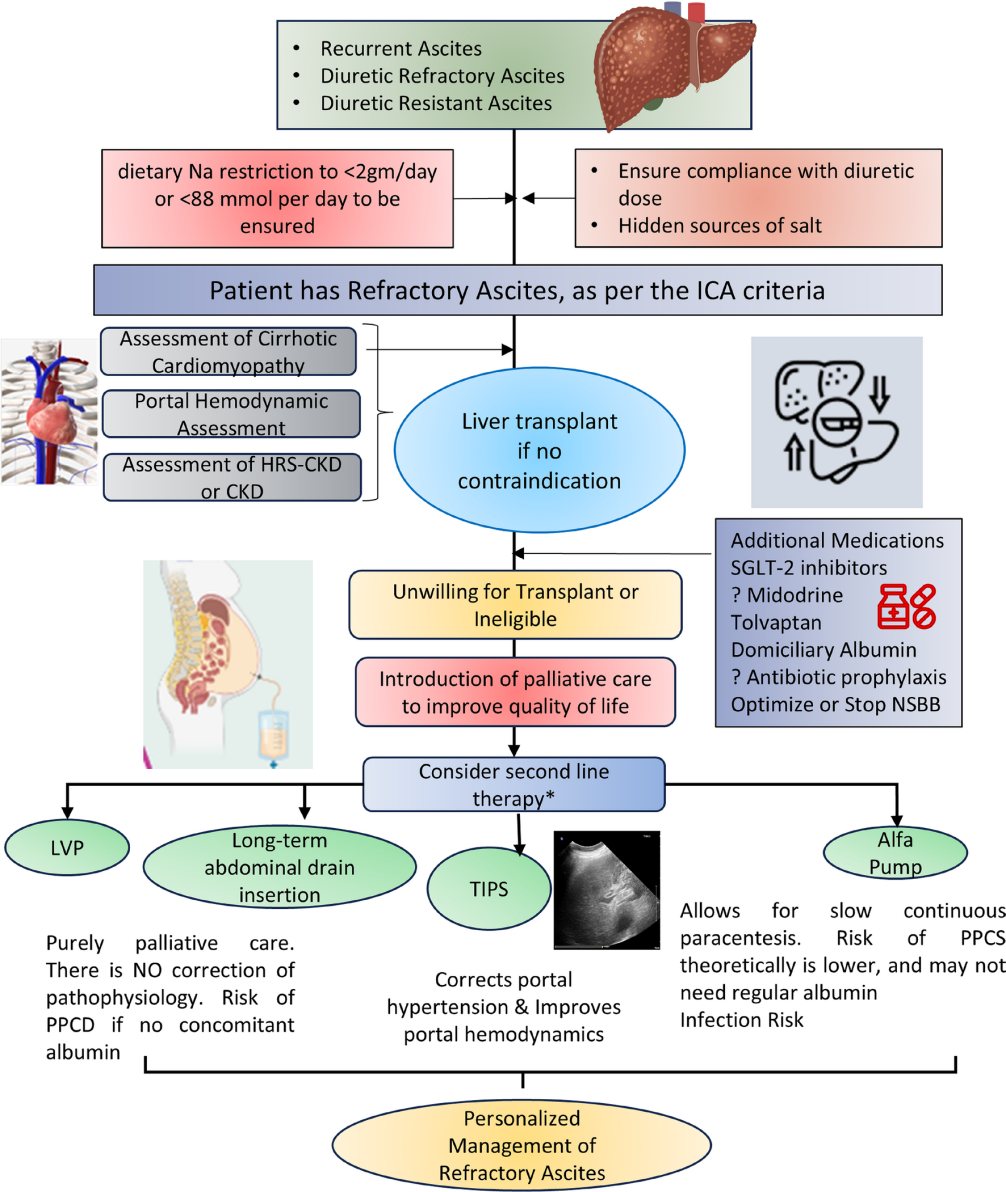
Algorithm for management of refractory ascites.

While LTAD provides clear benefits for patients and carers, it raises broader questions about the factors influencing HRQoL in end‐stage liver disease. LTADs are only one part of the solution; addressing the wider challenges faced by these individuals remains an ongoing issue. Therefore, the current study adds to resolving the clinical conundrum faced by clinicians who care for patients with RA, who are not eligible for LT or TIPS. It may offer a marginal benefit over frequent LVP in a small subset of individuals.

## Conflicts of Interest

M.P. serves as an associate editor of JGHOpen and was blinded to the peer review and editorial decision of this manuscript. The author declares no conflicts of interest.

## Data Availability

The data that support the findings of this study are available on request from the corresponding author. The data are not publicly available due to privacy or ethical restrictions.
